# Ultrasensitive Multi-Functional Flexible Sensors Based on Organic Field-Effect Transistors with Polymer-Dispersed Liquid Crystal Sensing Layers

**DOI:** 10.1038/s41598-017-02160-x

**Published:** 2017-06-01

**Authors:** Myeonghun Song, Jooyeok Seo, Hwajeong Kim, Youngkyoo Kim

**Affiliations:** 1 Organic Nanoelectronics Laboratory and KNU Institute for Nanophotonics Applications, Department of Chemical Engineering, School of Applied Chemical Engineering, Kyungpook National University, Daegu, 41566 Republic of Korea; 2 Priority Research Center, Research Institute of Advanced Energy Technology, Kyungpook National University, Daegu, 41566 Republic of Korea

## Abstract

Ultrasensitive flexible sensors with multi-sensing functions are required for various applications in flexible electronics era. Here we demonstrate flexible polymer-dispersed liquid crystal (PDLC)-integrated-organic field-effect transistors (OFETs) (PDLC-i-OFETs), which sensitively respond to various stimulations including weak gas (air) flow, direct physical touch, light, and heat. The flexible PDLC-i-OFETs were fabricated by spin-coating the poly(methyl methacrylate) (PMMA)-dispersed 4,4’-pentyl-cyanobiphenyl (5CB) layers on the poly(3-hexylthiophene) (P3HT) channel layers of OFETs with 200 μm-thick poly(ethylene naphthalate) (PEN) substrates. The flexible PDLC-i-OFET devices could sense very weak nitrogen gas flow (0.3 sccm), which cannot be felt by human skins, and stably responded to direct physical touches (0.6~4.8 g load). In addition, the present devices showed very sensitive photoresponses to a visible light and exhibited excellent heat-sensing characteristics at a temperature of 25~70 °C. In particular, the present flexible PDLC-i-OFET devices could sense two different stimulations at the same time, indicative of promising multi-sensing capabilities.

## Introduction

Sensors play a critical role in our daily life because they can detect various circumstances such as temperature change, gas leakage in kitchen or industry, brightness change in room or street, blood pressure change of human body, wind speed, etc.^1–3^. Such sensors are operated alone or embedded in various types of electronic systems (products)^4–6^. Most of conventional sensors are fabricated with inorganic materials so that they are generally hard and rigid leading to lack of softness and actual flexibility (bendability)^7–9^. However, recent paradigm change in electronics does actually drive electronic devices or systems to possess both flexible and lightweight features, as demonstrated in several mobile systems including smart phones or wearable smart watches/clothes^10–13^. In addition, multi-sensing functions are strongly requested for advanced sensors such as artificial skins for humanoid robots, protective electronic clothes for fire-fighters, integrated sensory modules for drones or automotive cars, etc^14–16^.

Considering such requirements, organic electronic devices are considered one of the promising candidates because organic (polymeric) materials can deliver high flexibility and lightweight features with a capability of bestowing various functions via chemical synthesis and blend technology^17–21^. Among several types of organic electronic devices, organic field-effect transistors (OFETs) have been intensively applied for various sensors because the transistor structure basically has a merit of amplifying signals due to their three electrode geometry^22–25^. In addition, the OFET sensors have a benefit in array type structures because each sensing cell in the matrix structure can be individually operated in the absence of interferences with nearby cells due to the principle of active matrix driving^26, 27^.

On this account, ultrasensitive tactile sensors have been developed by combining OFETs and liquid crystals (LCs) for possible applications to artificial skins of humanoid robots and wearable e-gloves or e-clothes etc^28–33^. The LC-integrated-OFETs (LC-i-OFETs), which can be divided into LC-on-OFETs and LC-g-OFETs, could detect an extremely low level of gas flows that could not be felt by human skins. However, the previous LC-i-OFET sensors have a drawback in terms of practical applications owing to the fully liquid state of LC in the sensing layers. In addition, no multi-functional sensing approach has been tried with the previous LC-i-OFET sensors.

In this work, we report multi-functional flexible PDLC-integrated-OFET (PDLC-i-OFET) sensors via successful preparation of stable solid-state LC sensing layers based on the concept of polymer-dispersed liquid crystal (PDLC). The PLDC sensing layers were prepared by embedding 4,4′-pentyl-cyanobiphenyl (5CB) micro-dots in the poly(methyl methacrylate) (PMMA) matrix, which were integrated on the flexible OFETs with 200 μm-thick poly(ethylene naphthalate) (PEN) substrates. The fabricated PDLC-i-OFET devices were able to sense four different objects such as weak air (gas) flow, strong physical force (touch), light, and heat.

## Results and Discussion

**Figure 1:**
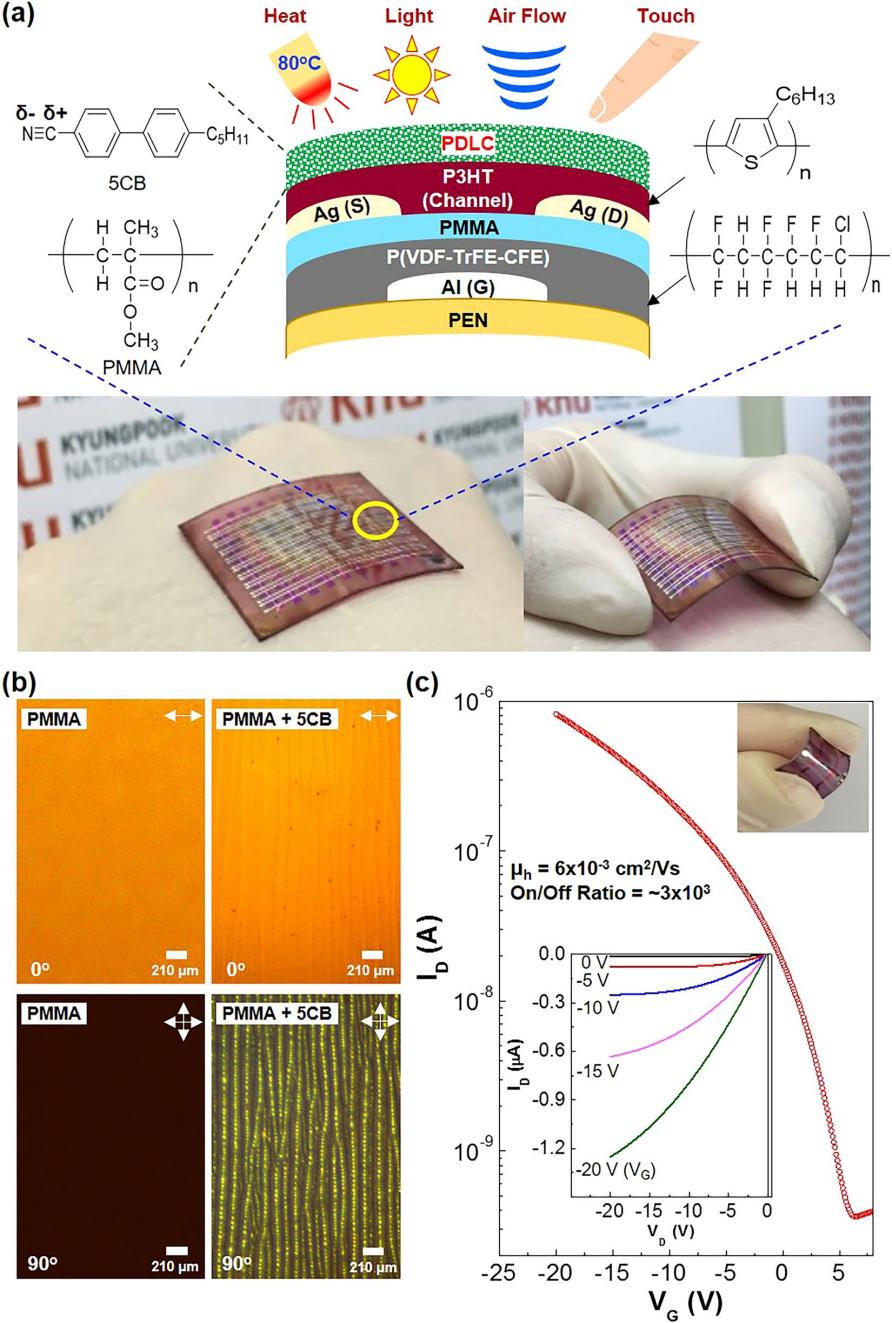
Structure, morphology, and basic device performances of flexible PDLC-i-OFETs. (**a**) Illustration for the device structure and materials used in this work (photographs demonstrate attachment of flexible PDLC-i-OFET array sensors on human hands). (**b**) Optical microscope images for the pristine PMMA layers (left) and the PDLC (5CB + PMMA) layer (right) under linear (0°) and cross (90°) polarization conditions. (**c**) Dark transfer curve (inset: output curves) for the flexible PDLC-i-OFET devices (V_D_ = -20 V).

As shown in Fig. 1a, the flexible multi-functional PDLC-i-OFET sensors were fabricated by spin-coating the PDLC sensing layers, which are composed of PMMA and 5CB, on the P3HT channel layers of the OFETs with the bilayer-type gate-insulating layers of poly(vinylidene fluoride-co-trifluoroethylene-co-chlorofluoroethylene) (P(VDF-TrFE-CFE)) and PMMA. The fabricated flexible PDLC-i-OFET sensors were easily bendable so that they were well fitted on the backside of human hands without any deformation (see bottom photographs in Fig. 1a). The morphology investigation with polarized optical microscopy (POM) revealed that the best composition was PMMA:5CB = 40:40 by weight because it could deliver relatively uniform and fine 5CB phases (see Figure S1). Looking into the PDLC layers by further increasing the magnification of the polarized microscope, a random line-like morphology was measured under linear-polarization condition (polarization angle = 0°) even though the pristine PMMA layers showed no particular morphology at the same linear-polarization condition (see Fig. 1b top). Interestingly, when the polarization angle was changed to 90° (cross-polarization condition), the random lines observed under linear-polarization condition became very clear and were found to consist of micro-dots with a diameter of ca. 30 μm (see Fig. 1b bottom and Figure S2). This result reflects that the micro-dots are composed of 5CB molecules of which orientation can change the polarization direction of light^34–36^. In particular, it was measured that the 5CB micro-dots were embedded inside the PMMA layers because putting a tissue paper on the PDLC layers didn’t give any wetting results (see Figure S3). As shown in Fig. 1c, the flexible PDLC-i-OFET devices exhibited typical p-type transistor behaviors (note that the device performance was slightly lowered by the deposition of the PDLC layers as compared in Figure S4a). A saturation behavior in drain current (I_D_) was measured from the output curves, while the drain current in the transfer curve was gradually increased with the gate voltage (V_G_) at a fixed drain voltage (V_D_). The on/off ratio and hole mobility of the flexible PDLC-i-OFET devices were ca. 0.3~1.6 × 10^4^ and ca. 1.3~6.0 × 10^−3^ cm^2^/V∙s, respectively (see Figure S4b). The stability test showed that the 5CB micro-dots were safely encapsulated by the PMMA domains without destruction even after seven days for storage with touch experiments (see Figure S5). In addition, a brief bending test showed that the device performance was quite well kept in the presence of marginal deviations even after 100 bending cycles (see Figure S6).

**Figure 2:**
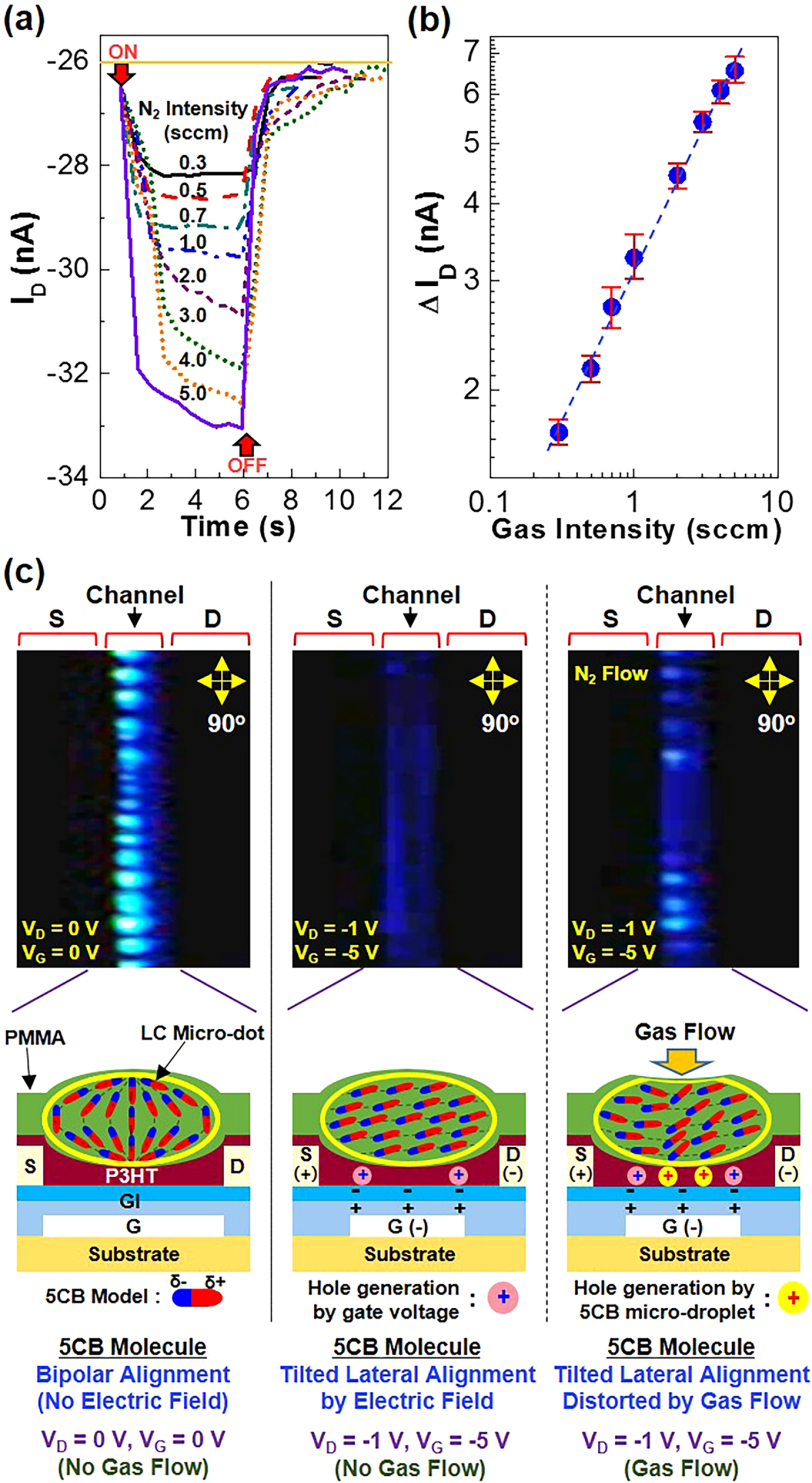
Sensing performances by weak gas flow stimulations and mechanism. (**a**) Drain current (I_D_) as a function of time under stimulation with nitrogen gas flows (gas intensity = 0.3~5.0 sccm for 5 s) for the flexible PDLC-i-OFET devices. (**b**) Drain current change (ΔI_D_) before and after nitrogen gas stimulations as a function of gas intensity (note that more than sixteen devices were measured). (**c**) Change of 5CB alignment in the channel region of PDLC-i-OFET devices according to applied voltages and nitrogen gas stimulations: (top) Polarized optical microscope images, (bottom) illustrations for possible orientation of 5CB molecules in the LC micro-dots in the channel layers.

First, the flexible PDLC-i-OFET devices were subject to the sensing test with very weak gas flow for proving the capability of sensing near field air environments. As shown in Fig. 2a, the drain current of devices was quickly increased upon stimulation with nitrogen gas flows of which intensity ranged from 0.3 sccm to 5 sccm. Interestingly, the drain current showed a level-off (stabilizing) trend under stimulation with nitrogen gas flow at a lower intensity, whereas it was further increased with a changed slope just before turning off at a higher gas intensity. Nonetheless, the net change in drain current (ΔI_D_) was quite linearly proportional to the nitrogen gas intensity (see Fig. 2b). This result may indicate that the alignment of 5CB molecules in the LC micro-dots of the PDLC sensing layers is very systematically sensitive to the intensity (strength) of nitrogen gas flow. In addition, the response of the present PDLC-i-OFET devices became much quicker than that of the previous devices with the fully liquid state LCs covered by a plastic film^31^.

To verify the change of 5CB alignment in the LC micro-dots, the channel regions of the flexible PDLC-i-OFET devices were investigated with the polarized optical microscope (Fig. 2c). Note that the direction of the LC micro-dot lines was controlled parallel to the channel length between source (S) and drain (D) electrodes. When the devices were turned off (V_G_ = V_D_ = 0 V), several bright ellipsoid-like micro-spots were measured in the channel region under cross-polarization condition. It is considered that these micro-spots correspond to the LC micro-dots containing 5CB molecules with an bipolar LC alignment (note that almost no light could be transmitted under cross-polarization if the 5CB molecules made a perfect homeotropic alignment)^28, 30, 36, 37^. Next, when the devices were turned on by applying voltages (V_G_ = −5 V and V_D_ = −1 V), the channel area became significantly darker in the presence of only very small portion of light transmitted under cross-polarization condition. This result indicates that most of the 5CB molecules in the LC micro-dots were basically aligned parallel to the source-to-drain direction due to the applied drain voltage (see the schematic diagram in the bottom-middle part of Fig. 2c). Considering the influence of negative gate voltage, each 5CB molecule in the LC micro-dots might make a slightly tilted lateral orientation in the channel region. When nitrogen gas flows were applied to the channel region, the channel region became bright again but randomly even though the brightness was relatively lower than the no bias (turn off) condition. This phenomenon can be attributed to the moving of 5CB molecules in the LC micro-dots upon stimulation with the nitrogen gas flow. Hence it is supposed that the nitrogen gas flow might distort the LC micro-dots leading to the alignment change of 5CB molecules (see the bottom-right part of Fig. 2c). As a result, the drain current could be negatively increased because most of 5CB molecules were affected to make their negative dipole ends contact the surface of the P3HT channel layers. As observed from Figure S7 (video clip), the brightness in the channel layer became dark again when the nitrogen gas flow was removed.

**Figure 3:**
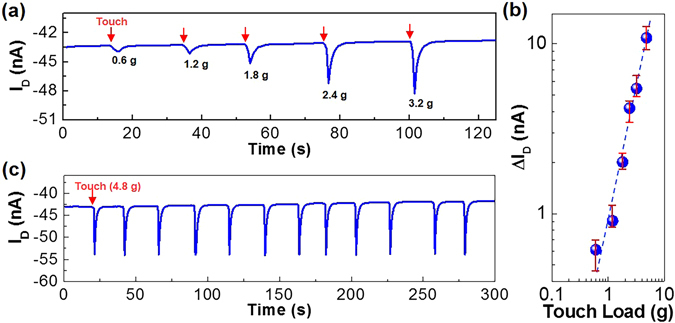
Sensing performances by direct physical touches. (**a**) Drain current (I_D_) response upon direct physical touch with various loads (0.6~3.2 g) from the flexible PDLC-i-OFET devices at V_D_ = −1 V and V_G_ = −5 V. (**b**) Net drain current change (ΔI_D_) as a function of physical load in (**a**) (note that more than sixteen devices were measured). (**c**) Drain current response upon repeated direct physical touches with 4.8 g load.

Then the direct physical touch test was performed by applying a pencil-like load on the surface of the PDLC layers in the flexible PDLC-i-OFET devices (V_G_ = −5 V and V_D_ = −1 V). As shown in Fig. 3a, touching with only 0.6 g load led to the negatively increased drain current in the presence of 2.3 s delay to reach the peak value. As the load strength (weight) increased, the peak current was gradually increased to the negative direction and the peak shape became much sharper with a steeper decay. This result provides following four-step mechanism: (1) The physical touch breaks the S-to-D direction orientation of 5CB molecules in the micro-dots of the PDLC layers; (2) The negative dipole ends of 5CB molecules change their orientation heading for the P3HT channel layer; (3) The negative dipole ends of 5CB molecules approach closely the P3HT layer after 2.3 s when it comes to the maximum (peak) drain current; (4) The 5CB molecules change their alignment to original positions (the state before the physical touch) when it comes to the drain current decay. This mechanism can be inferred from the schematic illustration in Fig. 2c, even though much more rigorous orientation changes of 5CB molecules in the micro-dots could be made by the stronger physical touches than the much weaker nitrogen gas flows. Interestingly, the drain current change (ΔI_D_) was linearly proportional to the strength of physical touch (see Fig. 3b), reflecting that the alignment change of 5CB molecules does linearly depend on the strength of physical touch. When 4.8 g load did repeatedly touch the surface of the PDLC layers, the drain current change was almost constant (see Fig. 3c). This result supports that the present flexible PDLC-i-OFET devices are quite stable in sensing and durable upon repeated touch stimulations.

**Figure 4:**
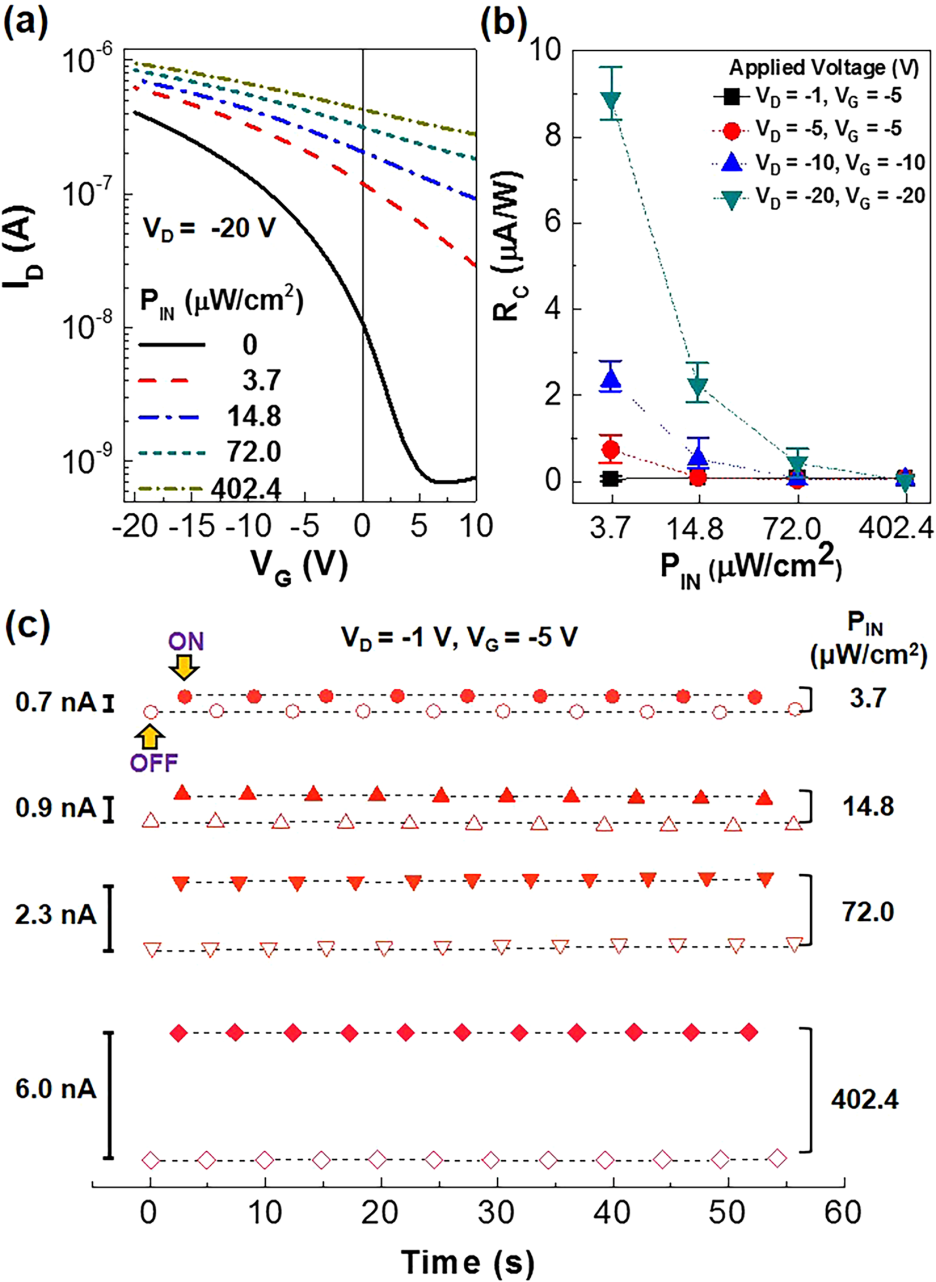
Photosensing performances under visible light (550 nm). (**a**) Change of transfer curves according to the incident light intensity (P_IN_) of green light (550 nm) for the flexible PDLC-i-OFET devices. (**b**) Corrected responsivity (R_C_) as a function of P_IN_ (note that more than sixteen devices were measured). (**c**) Stable drain current response by continuous operation of the flexile PDLC-i-OFET devices under visible light (550 nm) with different P_IN_.

Next, photosensing characteristics were examined by illuminating a monochromatic light (550 nm) to the P3HT channel layers through the PDLC layers in the flexible PDLC-i-OFET devices. As shown in Fig. 4a, the transfer curve at V_D_ = −20 V was significantly changed by the light illumination (see output curves in Figure S8). The drain current was gradually increased as the incident light intensity (P_IN_) increased, while the threshold voltage was largely shifted toward the positive gate voltage regions (see Figure S9). This result implies that the present flexible PDLC-i-OFET devices act as a photosensor, specifically a phototransistor when it comes to the photocurrent change controlled by the gate voltages^38–42^. Here it is noted that the P3HT part plays a key role in photosensing at the wavelength of 550 nm because of no optical absorption by the 5CB molecules^21, 43^. This fact supports that the lower photocurrent can be measured from the PDLC-i-OFETs compared to the OFETs without the PDLC layers at the same incident light intensity because of the smaller amount of P3HT in the PDLC layers than the pristine P3HT layers (see Figure S10). The corrected responsivity (R_C_), which corresponds to the ratio of net photocurrent (after subtracting dark current) to the incident light intensity, was higher at lower incident light intensities, indicative of the reduced exciton recombination at a lower light intensity (see Fig. 4b). As the gate and drain voltages increased, R_C_ was gradually increased owing to the improved collection of photo-generated charges. However, the R_C_ values for the present devices were relatively lower than those reported for simple (conventional) phototransistors because of the attenuated and/or scattering loss of incident light by the LC and/or micro-dots in the PDLC layers^40–42, 44^. As shown in Fig. 4c, the flexible PDLC-i-OFET devices exhibited stable photosensing characteristics under repeated light illumination irrespective of the incident light intensity.

**Figure 5:**
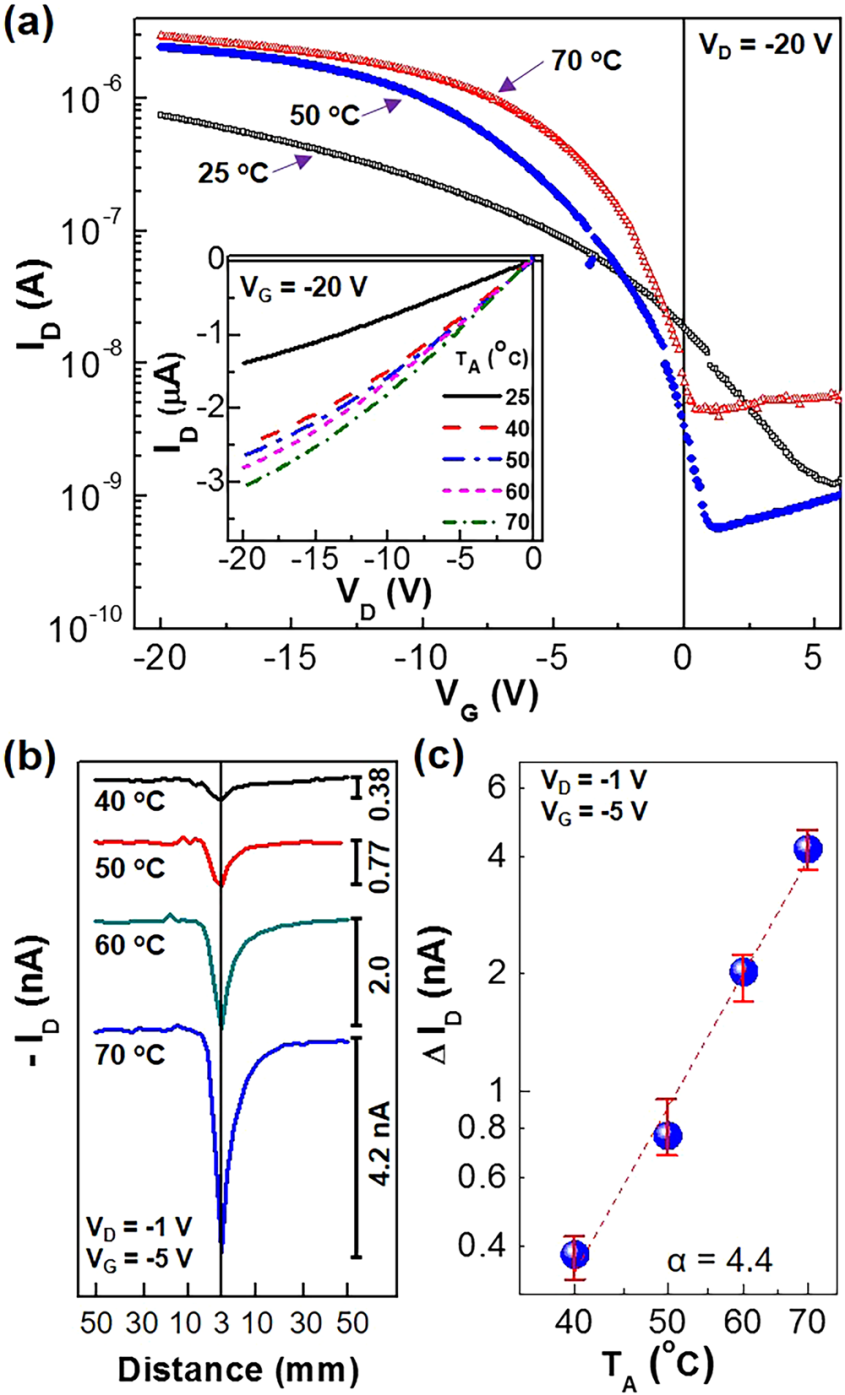
Thermal sensing performances under mild temperature conditions. (**a**) Transfer (inset: output) curves according to the temperature by heat sources (T_A_ = 25~70 °C) at V_D_ = −20 V and V_G_ = −20 V. (**b**) Drain current (I_D_) response according to the distance between the load-like heaters and the flexible PDLC-i-OFET devices. (**c**) Net drain current change (ΔI_D_) as a function of temperature (T_A_): The dashed line was fitted with the power-law equation (ΔI_D_ ~ T_A_^α^) (note that more than sixteen devices were measured).

Finally, the flexible PDLC-i-OFET devices were tested as a thermal (temperature) sensor by approaching a heat source toward the PDLC layers (note that the exact temperature was measured using a thermocouple according to the control distance of 3 mm). As shown in Fig. 5a, the transfer curves were noticeably shifted by the temperature changes. In general, as the temperature increased, the drain current of the transfer curves at higher gate voltages was found to be increased even though the base current at around turn-on (threshold) voltages was randomly changed (see Figure S11a). This trend is also supported from the output curves (see the inset graph in Fig. 5a and Figure S12). Here it is worthy to note that the present drain current variation according to the temperature can be mainly attributed to the change of charge transport in the P3HT parts, not by the change of 5CB phase (the nematic-to-isotropic temperature of 5CB = ca. 35 °C), when it comes to the linear correlation between the hole mobility and the temperature (see Figure S11b). As shown in Figure S13, both devices with and without the PDLC layers exhibited a noticeable change in transfer curves by the temperature variation from 25 °C to 75 °C. However, the shift direction of threshold voltage was different, which can be attributable to the dipole effect of 5CB in the PDLC layers of the PDLC-i-OFETs even though further investigation is necessary for clear understanding.

In order to examine the response time, the distance between the heat sources and the flexible PDLC-i-OFET devices was controlled to change between 3 mm and 50 mm. As shown in Fig. 5b, the drain current at V_G_ = −5 V and V_D_ = −1 V was quickly increased as the heat sources approached the PDLC layers of devices for all the temperatures tested in this work. When the heat sources were moved out of the devices, the fast decay in drain current was measured. Here it is worthy to note that the different response between approaching (rise time = ~2.2 s) and receding (decay time = ~8.4 s) of the heat sources can be attributable to the different thermal cycle of heating and cooling for the flexible PDLC-i-OFET devices. Interestingly, as shown in Fig. 5c, the change of peak drain current (ΔI_D_) as a function of approaching temperature (T_A_) was well fitted with a power-law type equation (ΔI_D_ ~ T_A_^α^, where α is an exponent).

**Figure 6:**
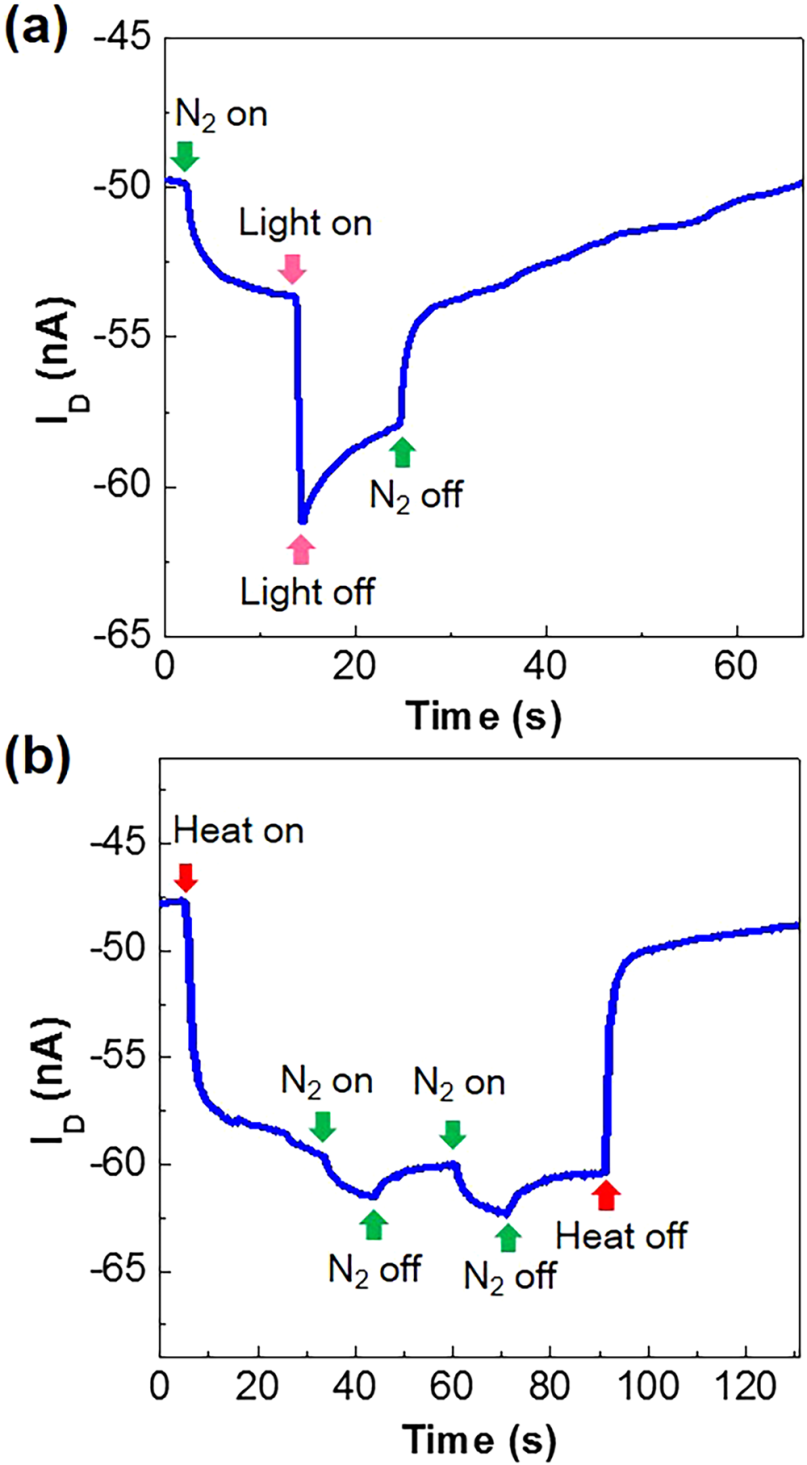
Multi-sensing performances. (**a**) Drain current (I_D_) response upon simultaneous stimulations with both nitrogen gas flow (5 sccm) and visible light (520 nm). (**b**) Drain current (I_D_) response upon simultaneous stimulations with heat (70 °C) and nitrogen gas flow (5 sccm). Note that applying sequences are different between (**a**) and (**b**).

In order to examine multi-sensing performances, two different stimulations were simultaneously applied to the flexible PDLC-i-OFET sensors. As shown in Fig. 6a, the drain current was first increased upon stimulation with the nitrogen gas flow (5 sccm for 10 s) and then it was further increased by illuminating with a green light (wavelength = 520 nm). As soon as the green light was turned off, the drain current was relatively slowly decayed and then further decreased by turning off the nitrogen gas flow. The relatively slow decay in drain current after turning off the green light can be attributed to the charging effects at the interfaces between the PDLC layers and the P3HT layers owing to the 5CB molecules with a high dipole moment (dielectric constant = ~11)^45, 46^. Next, both temperature and gas flow sensing were tested as shown in Fig. 6b. Applying the heat source (70 °C) first increased the drain current (negatively) and then further slight drain current increase was measured by applying the nitrogen gas flow (5 sccm) (note that the relatively small increase in drain current by the nitrogen gas flow can be ascribed to the weaker contribution of nitrogen gas flow than the temperature). Turning off the nitrogen gas flow reduced the drain current, which was quite reproducible when the continuous turn-on/off operation was performed. A close investigation finds that the response speed by the nitrogen gas flow was relatively slower at 70 °C than 25 °C (see Fig. 2a), which may be closely related to the isotropic phase of 5CB at 70 °C. When the heat source was removed, the drain current was largely decreased back to the baseline. These two individual tests confirmed that the present flexible PDLC-i-OFET devices are capable of multi-sensing even though the sensing objects is limited to four types such as weak air (gas) flow, direct physical touch, heat (temperature), and light at the moment. However, the selectivity for real multi-functionality is further required in order to perfectly distinguish each stimulation separately, even though the present devices demonstrate the possibility of simultaneous multi-sensing by combining the OFETs and the PDLC sensing layers.

## Conclusion

The multi-sensing flexible PDLC-i-OFET sensors, which exhibited typical p-type transistor behaviors, were fabricated by spin-coating the PDLC layers on the P3HT channel layers of OFETs. The PDLC layers were found to contain well-aligned LC micro-dots embedded in the PMMA layers. The nitrogen gas flow test revealed that the present devices could sense very weak gas flow (0.3 sccm), which cannot be felt by human skins, due to the orientation change of 5CB molecules inside the micro-dots upon gas stimulation. In addition, the flexible PDLC-i-OFET sensors exhibited stable and durable drain current responses upon the direct physical touch with pencil-like loads. The photosensing test confirmed that the present devices could sensitively respond to a visible light and exhibited stable photoresponses upon light modulation irrespective of light intensity. The thermal sensing test disclosed that the drain current change was well corresponded to the temperature change and highly reproducible upon approaching and receding of heat sources with different temperatures. Finally, the flexible PDLC-i-OFET devices could sense two different stimulations at the same time. Hence it is expected that the present concept of combining PDLC and OFET can contribute to further development of multi-functional organic sensory devices for various applications including humanoid robots, artificial skins, wearable sensors, etc.

## Methods

### Materials and solutions

P3HT (weight-average molecular weight = 30 kDa, polydispersity index = 1.7, regioregularity > 97%) were purchased from Rieke Metals (Lincoln, NE, USA). 5CB (purity = 98%) and PMMA (weight-average molecular weight = 120 kDa, polydispersity index = 2.2) were supplied from Sigma-Aldrich (USA), while P(VDF-TrFE-CFE) (weight-average molecular weight = ~500 kDa) was provided by Piezotech (France). The binary solutions of PMMA and 5CB were prepared using 2-butanone by varying the weight ratios (PMMA:5CB = 40:0, 40:10, 40:20, 40:40, and 40:60 by weight). The pristine P(VDF-TrFE-CFE) solutions were prepared using 4-methyl-2-pentanone at a solid concentration of 60 mg/ml, while the pristine PMMA solutions were prepared using chlorobenzene at a solid concentration of 20 mg/ml. These solutions were vigorously stirred at room temperature for 12 h prior to spin-coating. The P3HT solutions were prepared using toluene at a solid concentration of 15 mg/ml, which were stirred on a hot plate at 60 °C for 12 h before spin-coating.

### Thin film and device fabrication

Poly(ethylene naphthalate) (PEN) substrates (thickness = 200 μm) were prepared to make flexible sensor devices. The PEN substrates were subjected to wet cleaning with acetone and isopropyl alcohol in an ultrasonic cleaner for 30 min, followed by drying with nitrogen gas blows. After moving the cleaned PEN substrates into a vacuum chamber, 100 nm-thick Al layers were deposited on one side of the PEN substrates through a shadow mask to form gate electrodes. Then the P(VDF-TrFE-CFE) layers were spin-coated on the Al-deposited PEN substrates and thermally annealed on a hot-plate at 120 °C for 30 min. Next, the PMMA layers were spin-coated on the P(VDF-TrFE-CFE) layers and thermally annealed at 120 °C for 30 min. The P(VDF-TrFE-CFE)/PMMA bilayer-coated PEN substrates were transferred to a vacuum chamber and silver layers (Ag, 60 nm-thick) were deposited on the PMMA side of the bilayers through a shadow mask in order to make the source (S) and drain (D) electrodes at a base pressure of ~1 × 10^−6^ Torr. After taking out the S/D electrode-coated samples, the P3HT channel layers were spin-coated on the Ag-coated side of the samples and soft-baked on a hot-plate at 60 °C for 15 min. Finally, the PDLC sensing layers were formed on the P3HT channel layers by spin-coating using the binary solutions of PMMA and 5CB.

### Measurements

The thickness of films and electrodes was measured using a surface profiler (Alpha Step 200, Tencor Instruments). The transistor characteristics were measured using a semiconductor parameter analyzer (Model 4200SCS and 2636B, Keithley). The nitrogen gas flow sensing experiments were performed using a home-built sensor measurement system equipped with a probe station (PS-CPSN2, MODU-SYSTEMS), a micro-gas control module sets (TSC-210 and KRO-4000S, NF System), and a polarized optical microscope (FPG-30.2-4.3, CVI Melles-Griot). The direct physical touch test was carried out by dropping pencil-like loads (0.6~4.8 g) on the PDLC layers of devices (drop distance = 30 mm). The actual force was from 0.19 N/cm^2^ (0.6 g) to 1.52 N/cm^2^ (4.8 g). The photosensing performances were measured using a home-built photosensor measurement system, equipped with a monochromator (CM110, Spectral Products) and a light source (Tungsten-Halogen lamp, 150 W, ASBN-W, Spectral Products), which is connected to the semiconductor parameter analyzer units. A calibrated Si photodiode (818-UV, Newport, USA) was used for the accurate calculation of incident light intensity (P_IN_). A load-like heat generator was used for thermal sensing experiments, while the exact temperature was calibrated with a thermometer (62mini, Fluke). The distance between the heat generators and the devices was varied from 50 mm to 3 mm by utilizing a moving rail.

## Electronic supplementary material

Supporting Information

Video clip for Figure S3

Video clip for Figure S7
