# Supplementary material for: Ultrasensitive Multi-Functional Flexible Sensors Based on Organic Field-Effect Transistors with Polymer-Dispersed Liquid Crystal Sensing Layers
Source: Sci Rep. 2017 Jun 1;7:2630. doi: 10.1038/s41598-017-02160-x (PMC5453975; doi:10.1038/s41598-017-02160-x)
Supplement: Supplementary file 1 — Supporting Information [file 41598_2017_2160_MOESM1_ESM.pdf]

<Supplementary Information>

Ultrasensitive Multi-Functional Flexible Sensors Based on Organic Field-Effect Transistors with Polymer-Dispersed Liquid Crystal Sensing Layers

Myeonghun Song[1], Jooyeok Seo[1], Hwajeong Kim[1,2,*], and Youngkyoo Kim[1,*]

[1]Organic Nanoelectronics Laboratory and KNU Institute for Nanophotonics Applications, Department of Chemical Engineering, School of Applied Chemical Engineering, Kyungpook National University, Daegu 41566, Republic of Korea

[2] Priority Research Center, Research Institute of Advanced Energy Technology, Kyungpook National University, Daegu 41566, Republic of Korea

*Corresponding Authors: Email) ykimm@knu.ac.kr; khj217@knu.ac.kr

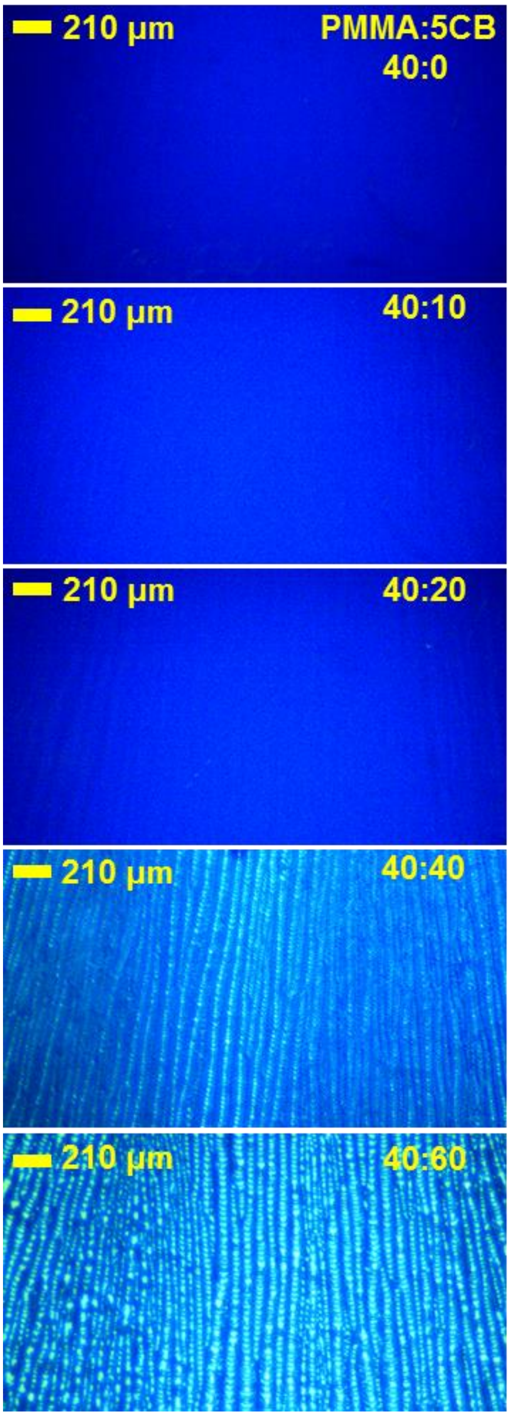

Supplementary Figure 1. Polarized optical microscope images (polarization angle = 90°) for the PDLC layers according to the weight ratio of PMMA and 5CB (by weight in mg unit) in order to choose the best composition for sensing layers. The 5CB micro-dots became pronounced as the content of 5CB increased, while their shape became irregular and bigger at the high content (PMMA:5CB = 40:60 by weight). Hence the composition of PMMA:5CB = 40:40 (by weight) was chosen for the PDLC sensing layers on the OFETs in this work.

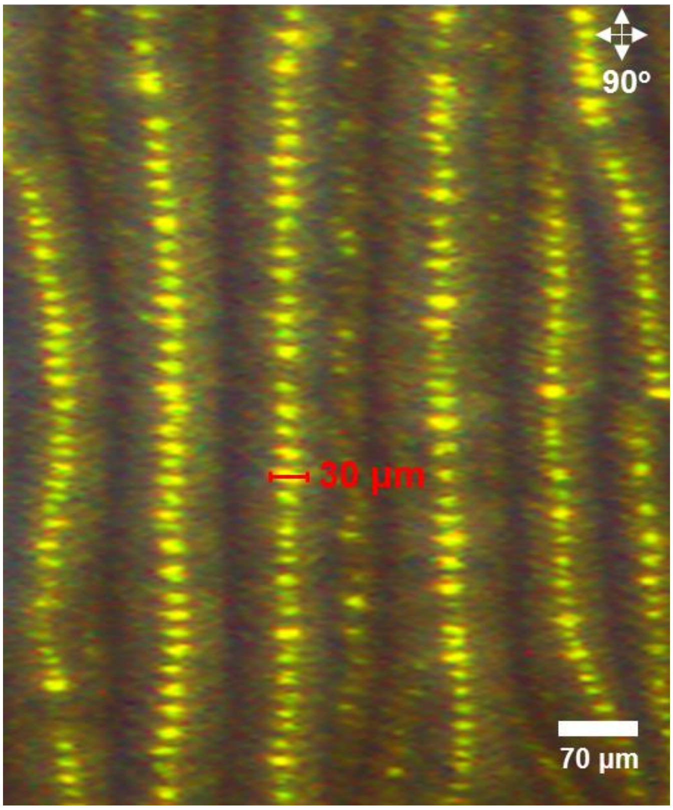

Supplementary Figure 2. The enlarged polarized optical microscope image (polarization angle = 90°) for the PDLC layers (PMMA:5CB = 40:60 by weight) coated on the P3HT layers. The size of 5CB micro-dots embedded in the PMMA layers was measured ~30 μm (see the red scale bar in the middle of the image).

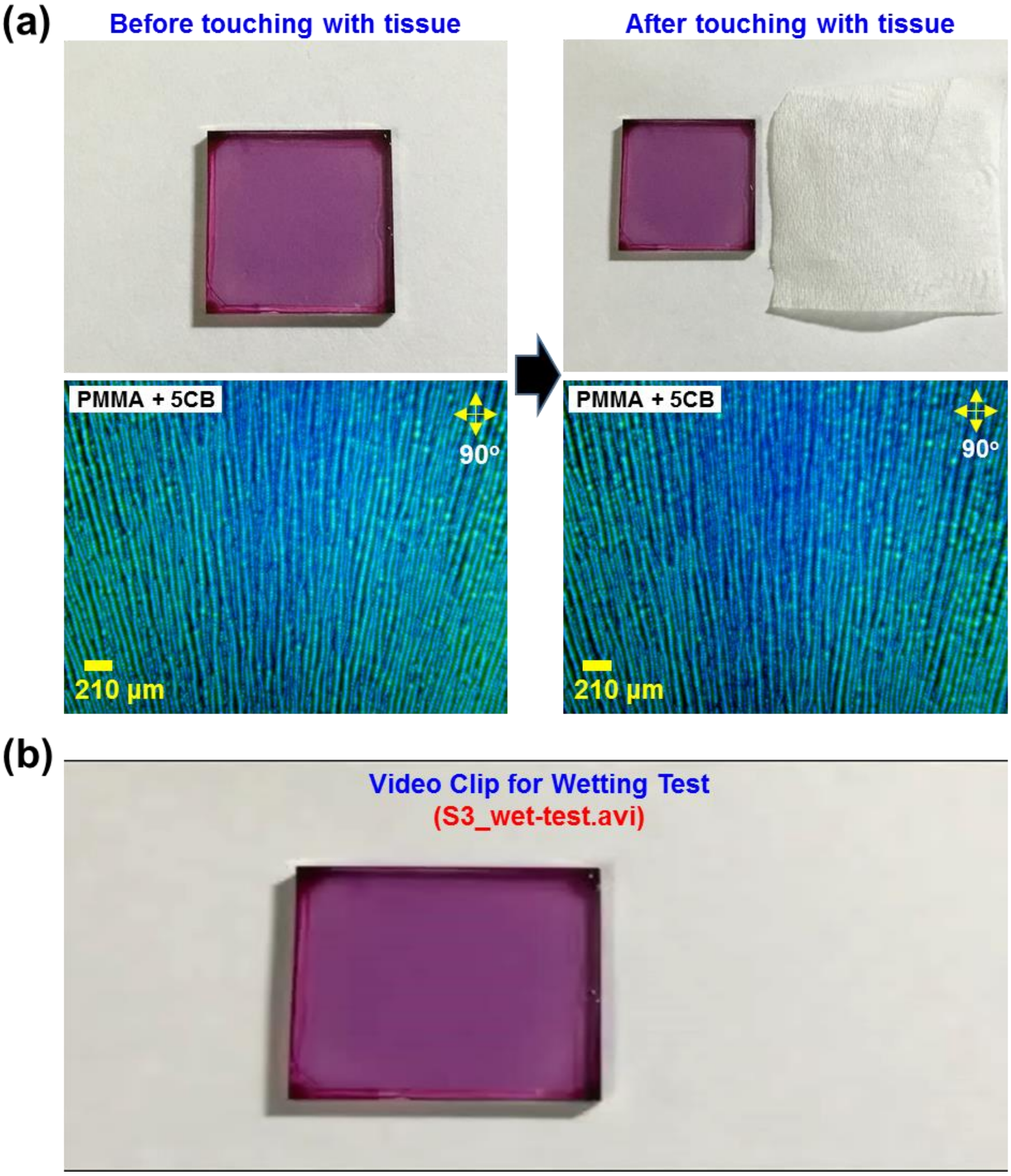

Supplementary Figure 3. Wetting test using a tissue paper in order to confirm whether the 5CB micro-dots were embedded completely in the PMMA layers (PMMA:5CB = 40:60 by weight): Note that ITO-glass substrates were used for the clear measurement of the morphology using the polarized optical microscope. (a) Before (left) and after (right) touching with the tissue paper. The bottom photographs in (a) show the polarized optical microscope images (cross-polarization condition) for the PDLC layers before and after touching with the tissue paper. Note that the micro-dot morphology was not changed after touching with the tissue paper leading to almost complete encapsulation of the 5CB micro-dots by the PMMA components, as can be clearly demonstrated from the video clip (b) (see touching with the tissue paper between 13 s and 21 s and the result at 25 s in the video clip).

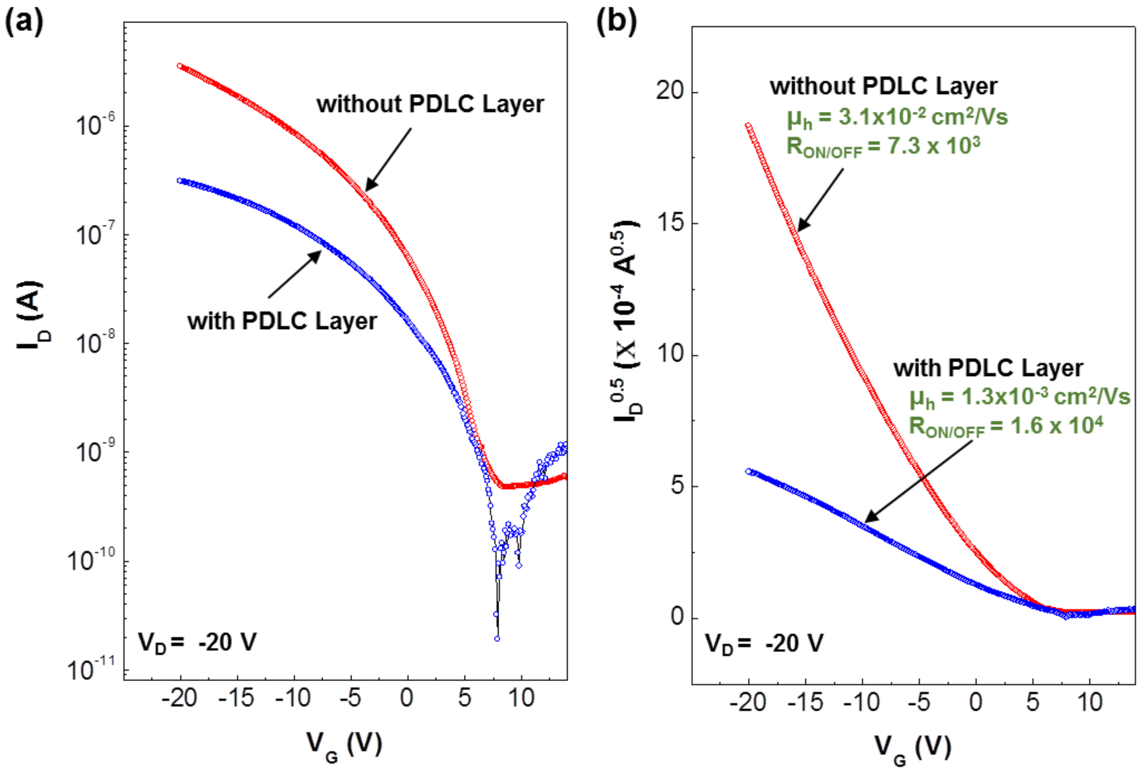

Supplementary Figure 4. Performance (dark) of OFETs with and without the PDLC layers (PMMA:5CB = 40:60 by weight): (a) Transfer curves, (b) $I_D^{0.5}$ – V_G plots. The hole mobility (μ_h) and on/off ratio ($R_{ON/OFF}$) are given on each plot. Note that the OFETs with the PDLC layers denote the PDLC-i-OFET devices.

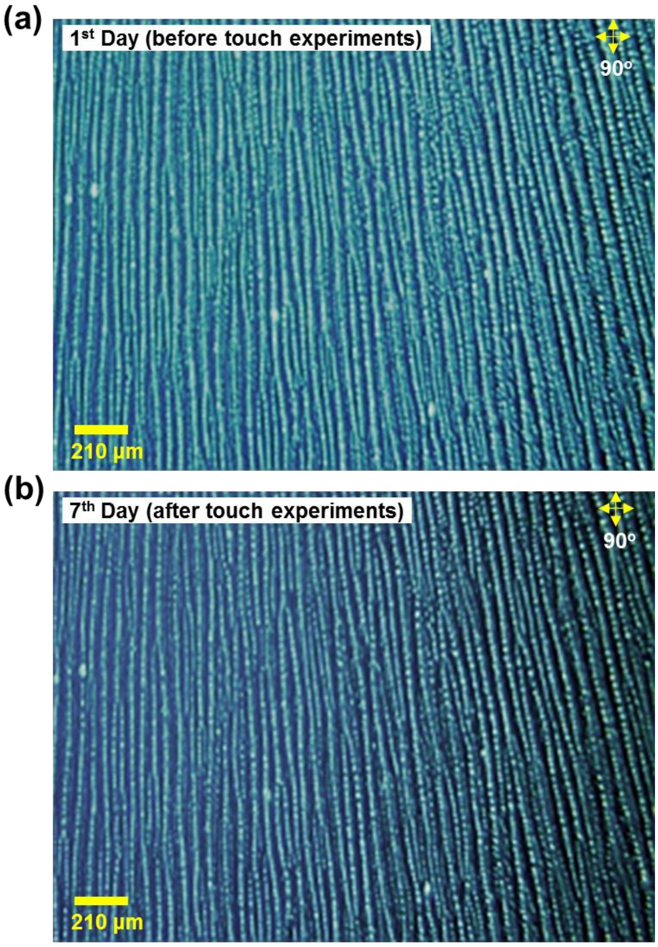

Supplementary Figure 5. Polarized optical microscope images (polarization angle = 90˚) for the PDLC layers (PMMA:5CB = 40:60 by weight) before (a) and after (b) touch experiments for 7 days. Note that the 5CB micro-dots were safely maintained without breaking even after touch experiments for 7 days.

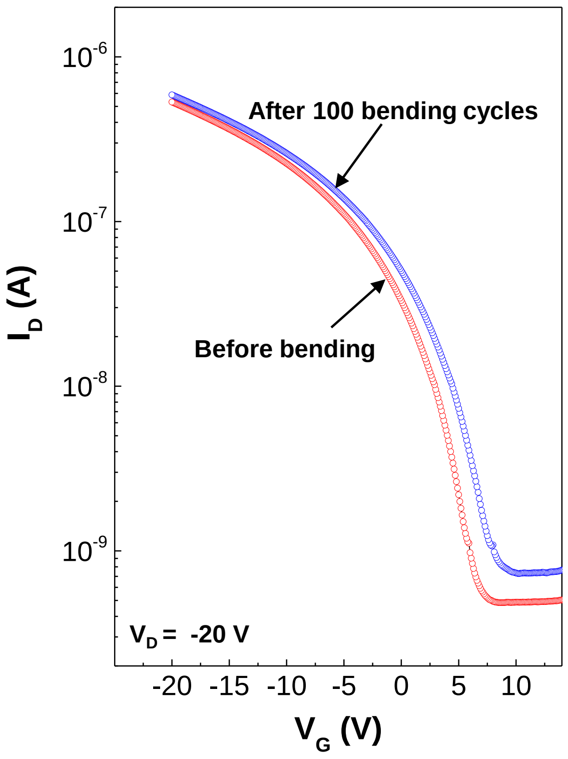

Supplementary Figure 6. Transfer curves (dark, V_D = -20 V) for the flexible PDLC-i-OFET devices before (red circles) and after 100 bending cycles (blue circles). Note that the slightly increased drain current after 100 bending cycles can be attributed to the increased off-current owing to the marginal degradation inside devices during bending.

Video Clip for Nitrogen Gas Sensing Test

(S7_gas-test.avi)

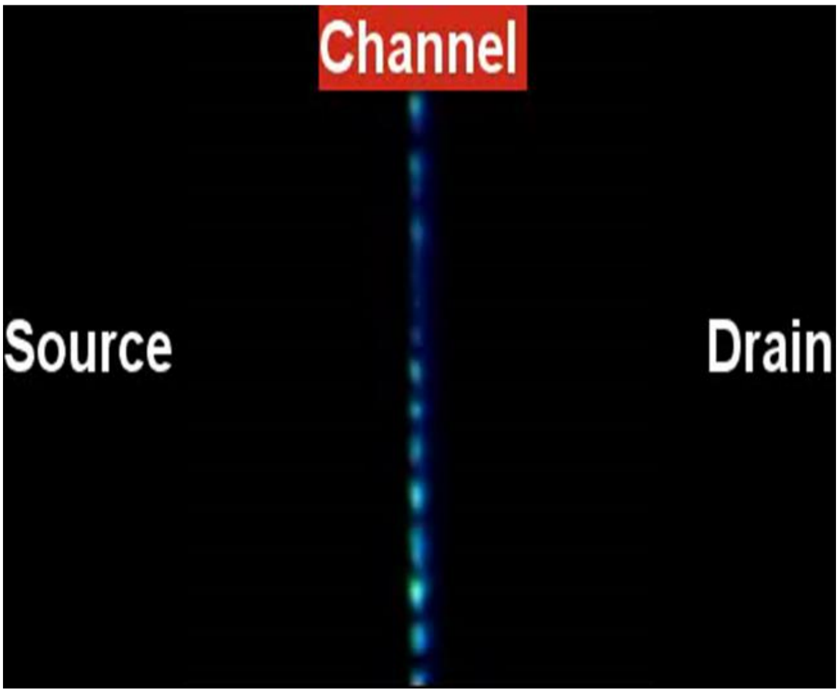

Supplementary Figure 7. Video clip (filename: S7_gas-test.avi) for the operation (cross-polarization condition) of the PDLC-i-OFET devices under stimulation with nitrogen gas flow (5 sccm, 83.3 μl/s). The applied voltages were V_D = -1 V and V_G = -5 V.

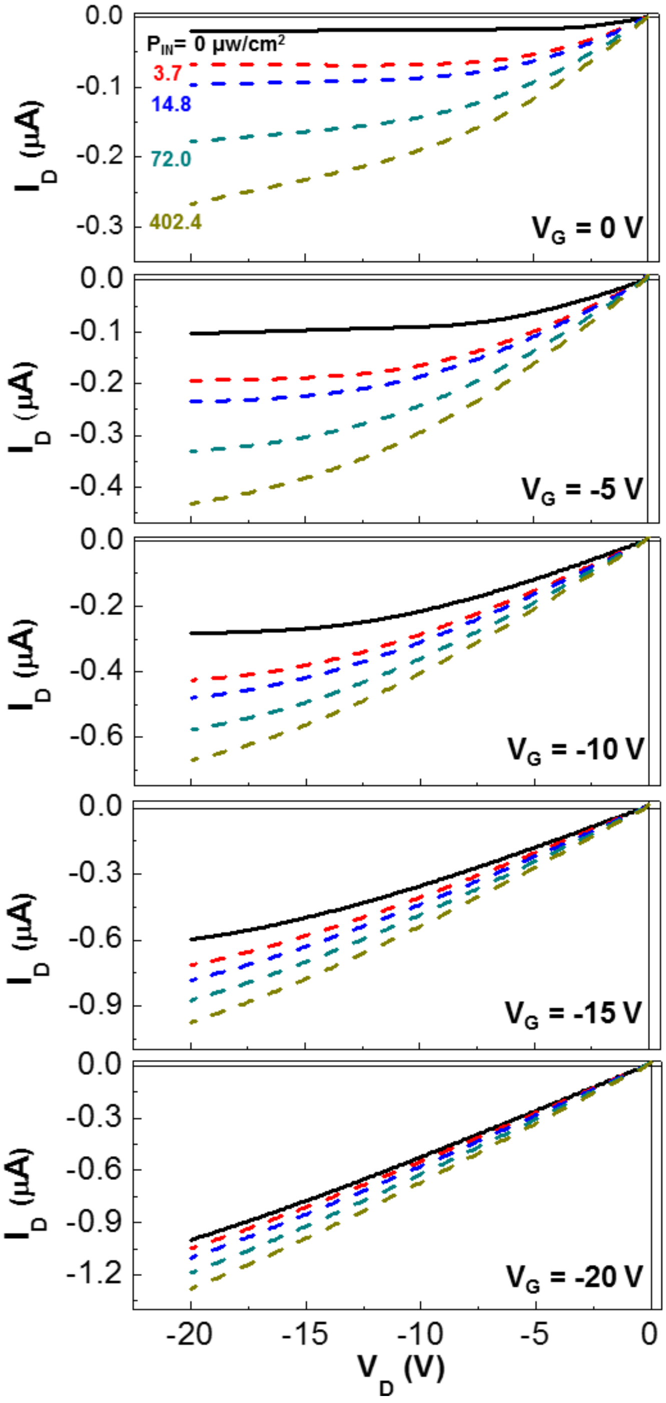

Supplementary Figure 8. Output curves according to the incident light (P_{IN}) and the gate voltage (V_G) for the flexible PDLC-i-OFET devices under illumination with a visible light (550 nm).

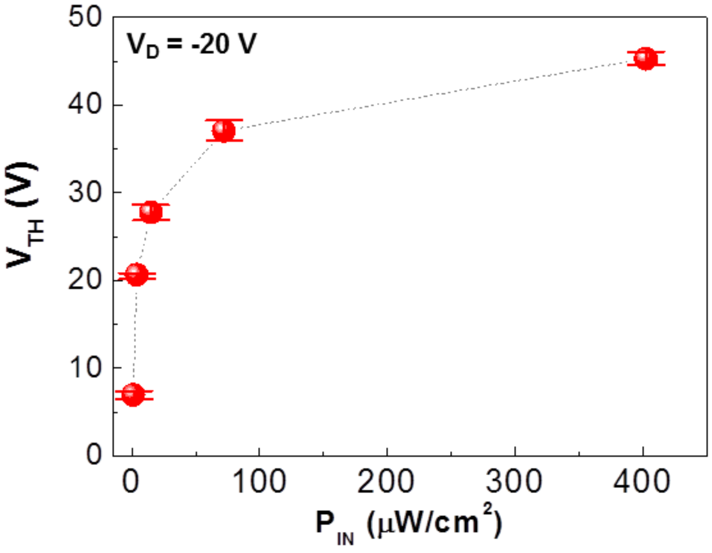

Supplementary Figure 9. Threshold voltage (V_{TH}) as a function of the incident light intensity (P_{IN}) for the flexible PDLC-i-OFET devices under illumination with a visible light (550 nm).

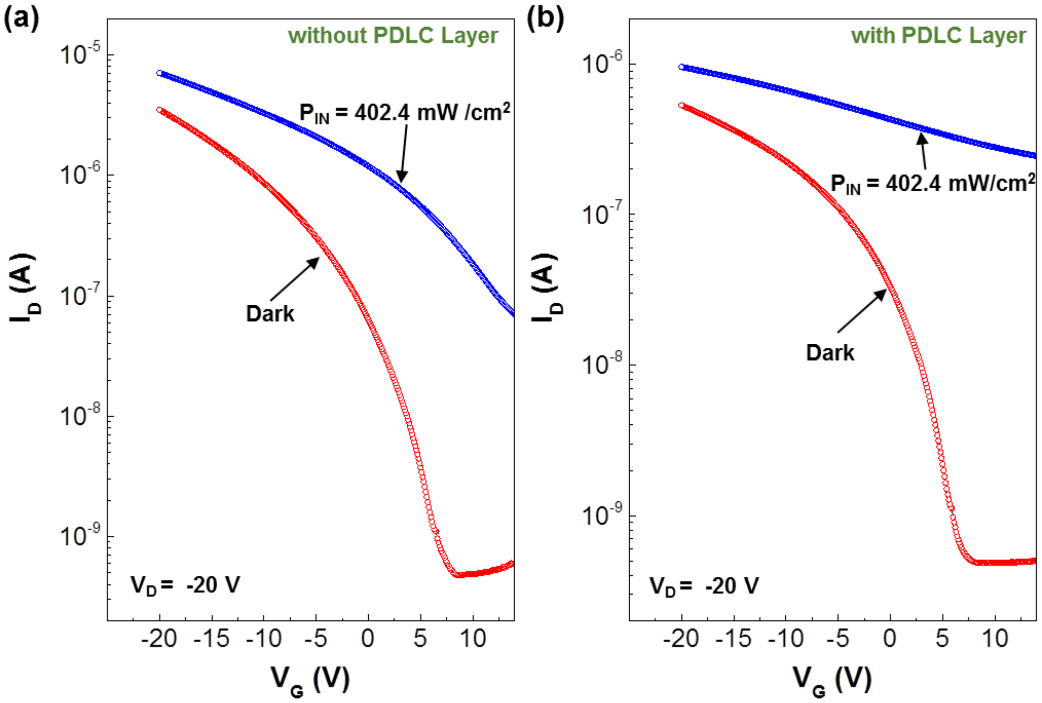

Supplementary Figure 10. Change of transfer curves before (dark) and after illumination with the green light (550 nm, P_{IN} = 402.4 mW/cm^2): (a) OFET without the PDLC layer, (b) OFET with the PDLC layer (PDLC-i-OFET).

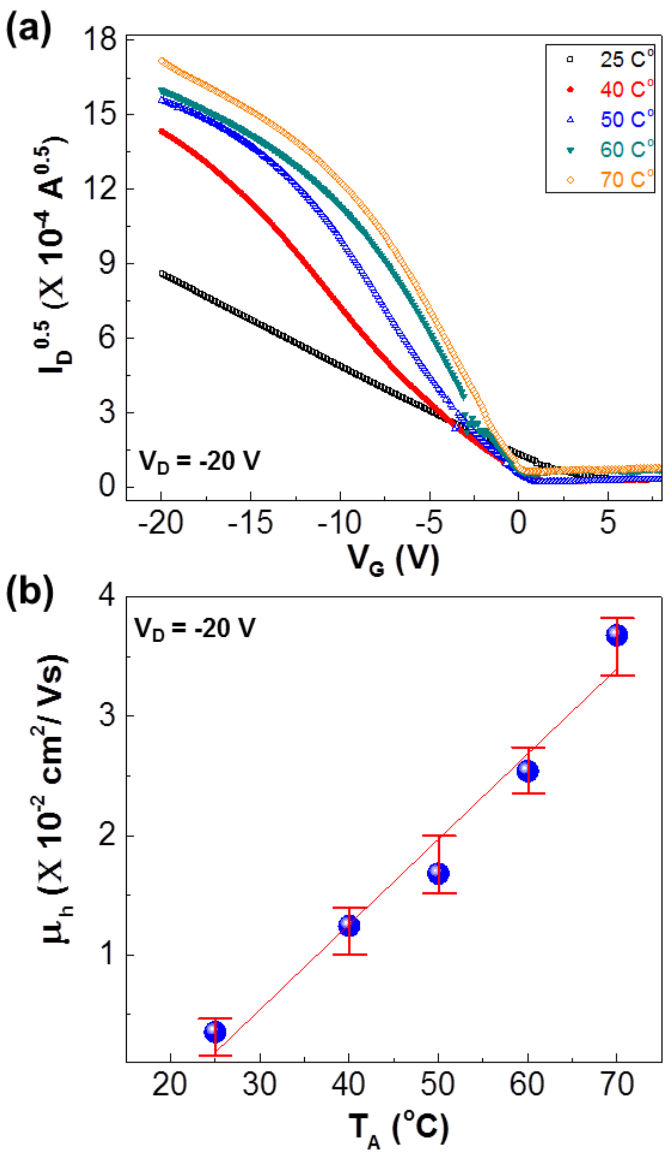

Supplementary Figure 11. (a) Square root of drain current ($I_D^{0.5}$) as a function of gate voltage (V_G) for the flexible PDLC-i-OFET devices at different temperatures by approaching corresponding heat sources. (b) Hole mobility (μ_h) as a function of temperature (T_A).

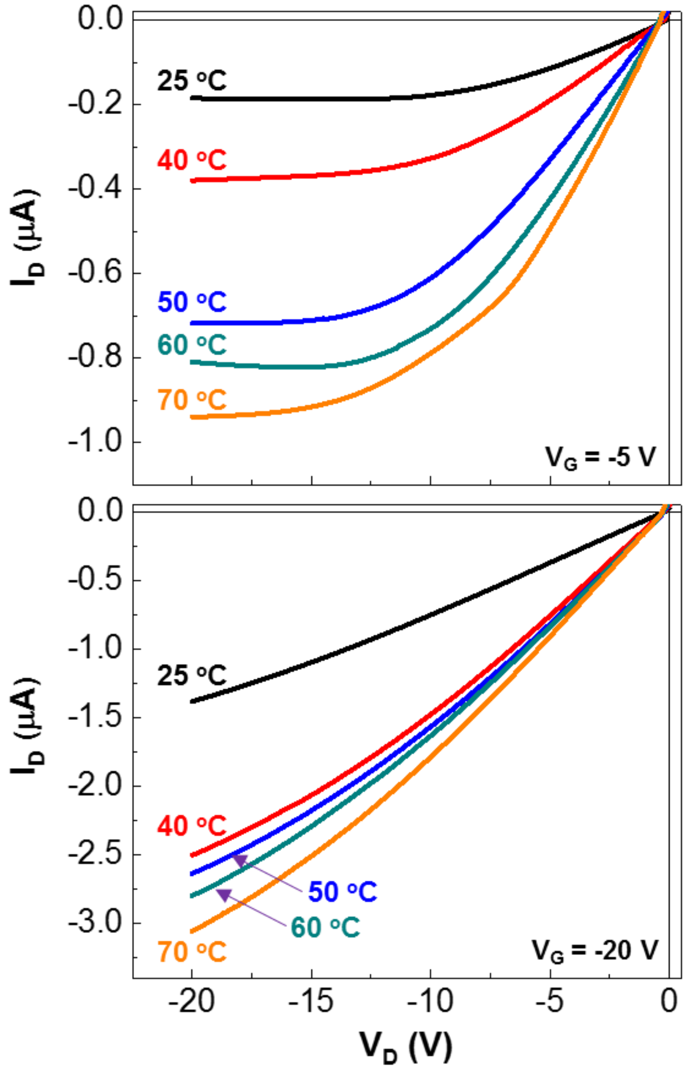

Supplementary Figure 12. Output curves at V_G = -5 V (top) and V_G = -20 V (bottom) for the flexible PDLC-i-OFET devices at different temperatures by approaching corresponding heat sources.

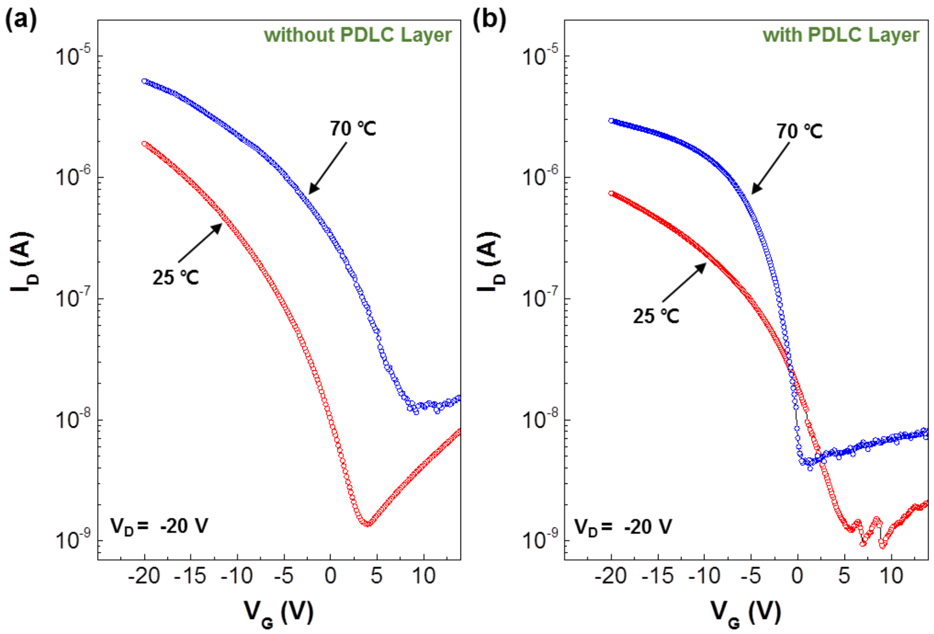

Supplementary Figure 13. Change of transfer curves at two different temperatures (25 ºC and 70 ºC): (a) OFET without the PDLC layer, (b) OFET with the PDLC layer (PDLC-i-OFET).
